# Multiscale design of cell-free biologically active architectural structures

**DOI:** 10.3389/fbioe.2023.1125156

**Published:** 2023-03-28

**Authors:** G. Ho, V. Kubušová, C. Irabien, V. Li, A. Weinstein, Sh. Chawla, D. Yeung, A. Mershin, K. Zolotovsky, L. Mogas-Soldevila

**Affiliations:** ^1^ Department of Bioengineering, University of Pennsylvania, Philadelphia, PA, United States; ^2^ Department of Graduate Architecture, DumoLab Research, Stuart Weitzman School of Design, University of Pennsylvania, Philadelphia, PA, United States; ^3^ Department of Architecture and Design, Slovak University of Technology, Bratislava, Slovakia; ^4^ Label Free Research Group, Center for Bits and Atoms, Massachusetts Institute of Technology, Cambridge, MA, United States; ^5^ Spatial Dynamics Program, Division of Experimental and Foundational Studies, Rhode Island School of Design, Providence, RI, United States

**Keywords:** interactive biomaterials, programmable matter, cell-free systems, biointeractive architecture, biodesign, biofabrication, material-driven design, additive manufacturing

## Abstract

Cell-free protein expression systems are here combined with 3D-printed structures to study the challenges and opportunities as biofabrication enters the spaces of architecture and design. Harnessing large-scale additive manufacturing of biological materials, we examined the addition of cell-free protein expression systems (“TXTL” i.e., biological transcription-translation machinery without the use of living cells) to printed structures. This allowed us to consider programmable, living-like, responsive systems for product design and indoor architectural applications. This emergent, pluripotent technology offers exciting potential in support of health, resource optimization, and reduction of energy use in the built environment, setting a new path to interactivity with mechanical, optical, and (bio) chemical properties throughout structures. We propose a roadmap towards creating healthier, functional and more durable systems by deploying a multiscale platform containing biologically-active components encapsulated within biopolymer lattices operating at three design scales: (i) supporting cell-free protein expression in a biopolymer matrix (microscale), (ii) varying material properties of porosity and strength within two-dimensional lattices to support biological and structural functions (mesoscale), and (iii) obtaining folded indoor surfaces that are structurally sound at the meter scale and biologically active (we label that regime macroscale). We embedded commercially available cell-free protein expression systems within silk fibroin and sodium alginate biopolymer matrices and used green fluorescent protein as the reporter to confirm their compatibility. We demonstrate mechanical attachment of freeze-dried bioactive pellets into printed foldable fibrous biopolymer lattices showing the first steps towards modular multiscale fabrication of large structures with biologically active zones. Our results discuss challenges to experimental setup affecting expression levels and show the potential of robust cell-free protein-expressing biosites within custom-printed structures at scales relevant to everyday consumer products and human habitats.

## 1 Introduction

Proponents of integration of synthetic biology tools such as DNA programming (Sato et al., 2022) into everyday materials offer exciting applications ranging from health and wellbeing to energy savings in the built environment (Beckett, 2021; Heveran et al., 2020). Following a “build it to understand it” philosophy, we here examine this compelling suggestion and study the obstacles to practicality of its transformative potential. We attempt a reduction to practice in an application specifically tailored for architecture. Synthetic biology and biological materials have so far progressed somewhat separately (Liu et al., 2022) here we consider embedded synthetic interactivity in biological material blends that are compatible with additive manufacturing (e.g., 3D-printing) and that could inform integrating biochemical material interactivity into large scale applications. There are many advantages of using biomaterials in conjunction with additive manufacturing: they are fully compostable, renewable, and many reduce the amount of energy during fabrication as they can be extruded and cured at ambient conditions. Several can be produced from waste of other industries such as food and agriculture, most can be architected to possess excellent mechanical properties and tunable structures (Mogas-Soldevila and Oxman, 2015; Mogas-Soldevila et al., 2014; Matzeu et al., 2020; Sanandiya et al., 2018). Biomaterials are also excellent candidates to host and enhance designed interactivity due to their high water content and customization properties (Nguyen et al., 2018; Zolotovsky, Gazit, and Ortiz, 2018; Fernandez and Dritsas, 2020; Matzeu et al., 2020). This work features the combination of large-scale biofabrication and cell-free systems which we conducted to examine the practical obstacles towards new applications of living materials out of the lab, beyond tissue engineering and drug delivery, and into architecture and design to create healthier, functional, sustainable, consumer-facing systems.

Biodegradable structures that include interactive components encoded by DNA are promising new directions for industrial design as they can be made to interact with the environment, sense it, and help diagnose it in strategies intuitive to humans. This is by means of biochemical reactions programmed to emerge during use and at end-of-life with regenerative capabilities. However, using living cells for this purpose presents many challenges, such as high complexity, extensive maintenance, low yields of proteins, and difficulty of standardization (Hong et al., 2022; Dixon and Kuldell, 2011; Huang et al., 2018). To address these challenges, cell-free systems are being developed for high-rate and high-yield protein expression for biological interactivity (Rolf et al., 2019; Dondapati et al., 2020; Nguyen et al., 2021; Hong and Serratosa Fernandez-Baca, 2022). Recently, researchers are advancing the robustness of cell-free systems and their stability under a wide range of conditions (Wilding et al., 2019; Yang et al., 2021) and for higher protein yield and optimized storage (Smith et al., 2014; Das Gupta et al., 2022). Modular platforms have been developed to expand the range of applications of cell-free systems into applications meant to operate in the often harsh and unpredictable conditions of the real-world (Tourlomousis et al., 2019; Garenne et al., 2021; Das Gupta et al., 2022).

Cell-free transcription-translation (TXTL) relies commonly on a cytoplasmic extract that provides the molecular components necessary to recapitulate gene expression *in vitro*, and while a wide variety of platforms exists derived from various organisms including mammals, most of the commercially available systems are derived from *Escherichia coli.* (Garamella et al., 2019). Previous research showed initial evidence for compatibility of cell-free reactions in bioblends. Whitfield et al. demonstrated mCherry cell-free expression in macro-scale hydrogel materials. In these experiments cell-free reactions were lyophilized and rehydrated in a range of hydrogel materials. This resulted in increased protein production in some gels (Whitfield et al., 2020). Lee et al. showed that the addition of silk protein to cell-free reactions in solution improves both protein productivity and kinetics (Lee et al., 2020). Volume exclusion and shielding properties could be the benefits of silk fibroin creating a macromolecular entropic crowding effect, similar to the study (Ge et al., 2011) showing macromolecular additives achieving increases in cell-free activity in several, -but not all-cases. That and other evidence for cell-free functional protein synthesis in biopolymer matrices (Lee et al., 2020; Whitfield et al., 2020) motivated our research in the practicality of bio-active sites within printed bioblends for biologically programmed architectural structures.

Organisms use materials available in their environment to build sometimes large, functional structures such as wood, skin, leafs, or bone (Ortiz and Boyce, 2008). Since recent advances in additive manufacturing, their mechanics of assembly are being better understood and emulated in man-made computational and fabrication systems. For instance, researchers 3D print multiscale hierarchical structures with layered compositions of materials of varying properties or determine graded performance in stiffness, flexibility, self-shaping, or decay (Bonderer et al., 2008; Cao et al., 2018; Li and Ortiz, 2015; Duro-Royo et al., 2018; Duro-Royo et al., 2015; Giachini et al., 2020; Yao and Ishii, 2019). To design and fabricate our structurally and biologically programmed structures, we use a meter-scale additive manufacturing platform that operates at ambient conditions. It is tuned to the rheological and chemical conditions of water-based biological material blends, simulates natural assembly *via* evaporation, and is continuously being upgraded from our previous work (Mogas-Soldevila et al., 2021).

**FIGURE 1:**
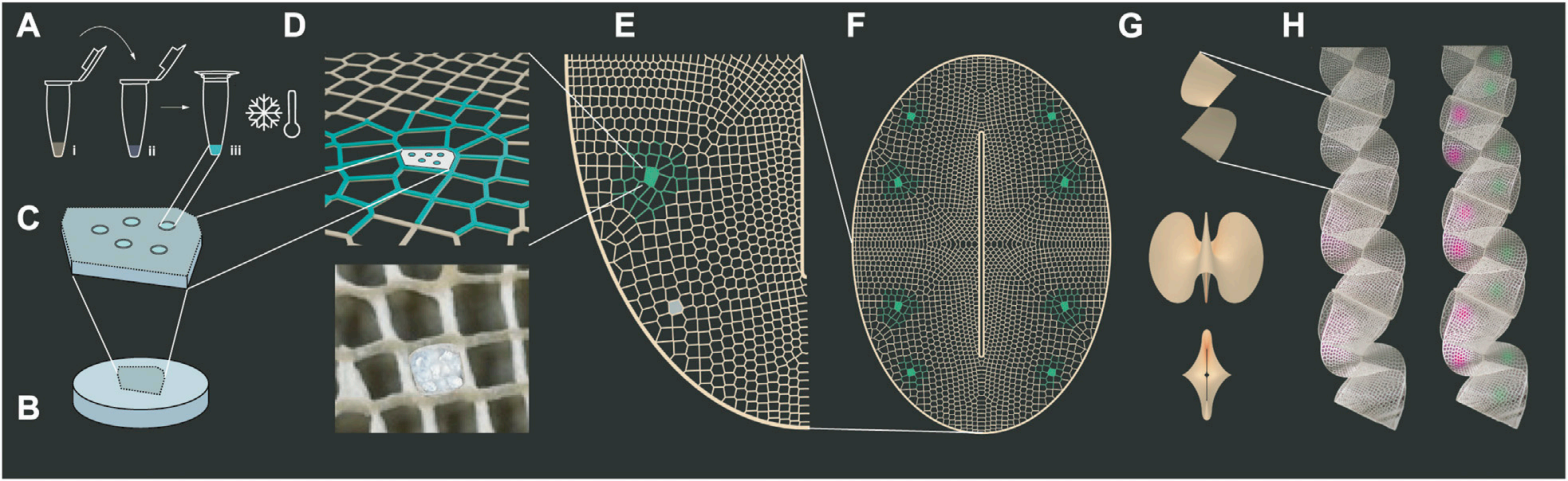
Multiscale Design for Bio-interactive Systems. Microscale design: **(A)** Cell-free reactions of commercial TXTL are added to biopolymers in diverse concentrations and freeze dried to test their expression after simple rehydration: (i) TXTL, Mastermix, and DNA composing the expression kit (ii) silk fibroin or sodium alginate biopolymer, (iii) lyophilization. **(B)** Biopolymer blend disks are made by lyophilization as mechanical support to host cell-free reactions in “A”. Mesoscale design: **(C)** Configurations of pebells “**A**” are encrusted into “**B**” to form biosites. **(D)** These are mechanically press-fitted into specific cells within additively manufactured 50 cm-long fibrous biopolymer lattices. **(E)** Certain lattice areas are conferred with higher sparsity and porosity to support biosite performance. Macroscale design: **(F)** An oval with a longitudinal slit is chosen as **(G)** a 2d-to3d folding modular shape able to conform into ribbons. **(H)** Ribbons are distributed as indoor surfaces or partitions and envisioned as air-interactive devices in our future work.

The work presented here derives a multiscale design platform for biointeractive systems. In particular, half-a-meter long biologically active biopolymer lattices are produced that operate at three scales as depicted in Figure 1: (a,b,c) supporting cell-free protein expression in a biopolymer matrix (micro-scale), (d,e,f) varying material properties of porosity and strength within two-dimensional lattices to support biological and structural functions (meso-scale), and (g,h) obtaining folded indoor surfaces that are structurally sound and biologically active (macro-scale).

As explained in our Results section, we embed commercially available cell-free protein expression systems with reporter green fluorescent protein (GFP) within silk fibroin and sodium alginate biopolymer matrices formed into bioactive pellets and use GFP fluorescence to confirm their compatibility. We demonstrate press-fitting of these freeze-dried bioactive pellets into a printed foldable fibrous biopolymer lattice. Our results show first steps towards modular multiscale fabrication of large structures with biologically active zones. We inform the consideration of opportunities and obstacles towards future application scales. These range from a few millimeters up to a few meters and from; everyday objects, upholstery, furniture, tiling, interior partitions, facade systems, or entire homes built with non-toxic and environmentally interactive biodegradable materials. This is of significant urgency as we seek to replace the many carcinogenic, carbon-positive and in other ways environment-polluting, unhealthy materials currently routinely used as the core structures, additives, and coatings in these application areas (Ashby, 2012).

## 2 Materials and methods

### 2.1 Preparation of TXTL-Biopolymer blends

#### 2.1.1 Biopolymers

All chemicals unless otherwise specified were purchased from Sigma Aldrich. Sodium alginate (SA) solution was prepared at different concentrations by mixing Pure Sodium Alginate food-grade powder from Modernist Pantry with water using a countertop blender with 1800 W power, hardened stainless-steel blade, running at up to 30,000 RPM, then left to off gas naturally in fridge. Silk fibroin (SF) solution was extracted from silk moth *Bombix mori* cocoons by Canon Virginia at 6% w/v final concentration and 135.22 kDa molecular weight.

#### 2.1.2 Cell-free reactions

All cell-free reactions were made using the myTXTL T7 Expression kit (Arbor Biosciences), using the included 0.1 nM P70a-T7rnap HP helper plasmid and 1 nM T7p14-deGFP HP reporter plasmid, except for the diluted TXTL-biopolymer mixes which used 5 nM of the provided GFP plasmid. All negative controls were created using the same conditions, apart from nuclease-free water in place of the provided deGFP plasmid. All incubations were done in PCR tubes in a T100 Thermocycler (Bio-Rad) for the kit manufacturer’s recommended optimal temperature of 27°C for 16 h followed by an indefinite hold at 4°C to halt expression until samples were retrieved, normally within the hour. Lyophilization of biopolymers and TXTL-biopolymer mixes in PCR tubes were performed in a small Harvest Right freeze dryer under an overnight freezing cycle and 6 h of sublimation.

#### 2.1.3 Preparation of diluted TXTL-Biopolymer blends

S30A buffer was prepared at a final composition of 14 mM Mg-glutamate, 60 mM K-glutamate, 50 mM Tris acetic acid pH 7.7. The effect of dilution compared to a reaction of myTXTL at standard concentrations in 3 µL volume was tested by creating a 1:1 dilution of myTXTL with either S30A buffer, 6% SF, or 1% SA (6 µL). Triplicates of each condition were then incubated at 27°C for 16 h followed by an indefinite hold at 4°C to halt expression until samples were retrieved. For all conditions, phosphate buffered saline (PBS) was added up to 25 µL and then transferred to a 384-well plate for fluorescence to be measured.

#### 2.1.4 Preparation of undiluted TXTL-Biopolymer blends

Undiluted TXTL-biopolymer blends were prepared by first freeze drying corresponding amounts of biopolymer, then rehydrating in a standard TXTL reaction and mixing gently by pipetting. Triplicates of 3 µL for each condition were then incubated at 27°C for 16 h followed by an indefinite 4°C hold to halt expression until samples were retrieved. PBS was added up to 25 µL and then transferred to a 384-well plate for fluorescence to be measured.

#### 2.1.5 Preparation of lyophilized TXTL-biopolymer pellets

1% SF-TXTL and 0.25% SA-TXTL were prepared using the undiluted method in 3 µL volumes, then lyophilized again along with 3 µL samples of only TXTL. For each time point and condition, triplicates of freeze-dried pellets were kept covered at room temperature. To measure expression, pellets were rehydrated in 3 µL nuclease-free water and gently mixed by pipetting and agitation. These were incubated at 27°C for 16 h followed by a 4°C hold. PBS was added up to 25 µL and then transferred to a 384-well plate for fluorescence to be measured.

### 2.2 Characterization

Fluorescence was measured in a Tecan Infinite M200 Plate Reader in a black clear bottom 384-well plate (MatTek). All endpoint measurements were measured at *λ*_ex_ 460 nm with *λ*_em_ spectra from 495 to 800 nm. The settings were chosen due to a fixed 35 nm bandwidth of the plate reader and to confirm the characteristic spectra of GFP. The peak at 511 nm was used for subsequent analysis. Responses to ambient changes of the biosites were only tested under expression tests in PCR tubes in the thermocycler. The overall lattices are currently being subjected to sunlight, air temperature changes and humidity changes and effects have yet to be observed. We do know from previous research that these materials will ultimately decay and be able to compost, which is desired, but in indoor use as is the focus of this article, that might take years.

### 2.3 Fabrication

The printing process is the topic of upcoming work and publication aimed at characterizing printability of non-biologically-active blends and as such outside the scope of this article. Here we detail the incorporation and viability of cell-free pathways within our printable blends. Biological material lattices were computationally designed within Rhinoceros3D CAD platform and its parametric plugin Grasshopper, then fabrication instructions of synchronized positioning and deposition were sent to be additively manufactured with a pneumatic extrusion platform operating at ambient conditions. We used a 1.4 mm inner diameter nozzle which determined printing thickness in wet state. Printed paths’ width after drying was reduced to 1.2 mm, and their height to 0.9 mm. These details and more on the printed blends are the focus of an upcoming article -as mentioned-that will include rheology, yield stress characterization, tensile and 3-point bend testing, etc. Mechanical attachment of freeze-dried composites to dry printed lattices was performed by press-fitting of cut-to-size sponges to designated lattice cell cavities, optimizing fitting conditions will be a necessary step towards further scaleup.

## 3 Results: Multiscale design platform for bio-interactive systems

We reduce to practice a method for cell-free protein expression biotechnologies bridging DNA-scale design to meter-scale material substrates with programmable, life-like functions -absent of any living cells. These functions have been already studied and reported to include embedded sensing, energy generation and storage, self-repair, and self-actuation into man-made matter to attain what natural materials like skin and wood can do *via* cells living in association with a structural scaffold (Nguyen et al., 2018; Moser et al., 2019). Described below is a multiscale method integrating cell-free reactions in silk fibroin and sodium alginate biological materials creating large-scale printable bio-interactive material systems (Figure 1). “Bioblends” is here used to refer to polymer mixes of biological materials such as cellulose, chitosan, sodium alginate and silk fibroin.

### 3.1 Microscale Design Methodology

The goal at this scale is to achieve a structurally robust porous pebble with cell-free protein expressing mix (TXTL) embedded in the biopolymer matrix (Figures 1C,D). We are using cell-free reactions to express GFP to test the expression system within silk fibroin and sodium alginate biopolymers which present appropriate pH for cell-free applications and are increasingly used in experimental design and are traditional in biomedical applications.

#### 3.1.1 Biopolymer—TXTL: Dilution

**FIGURE 2:**
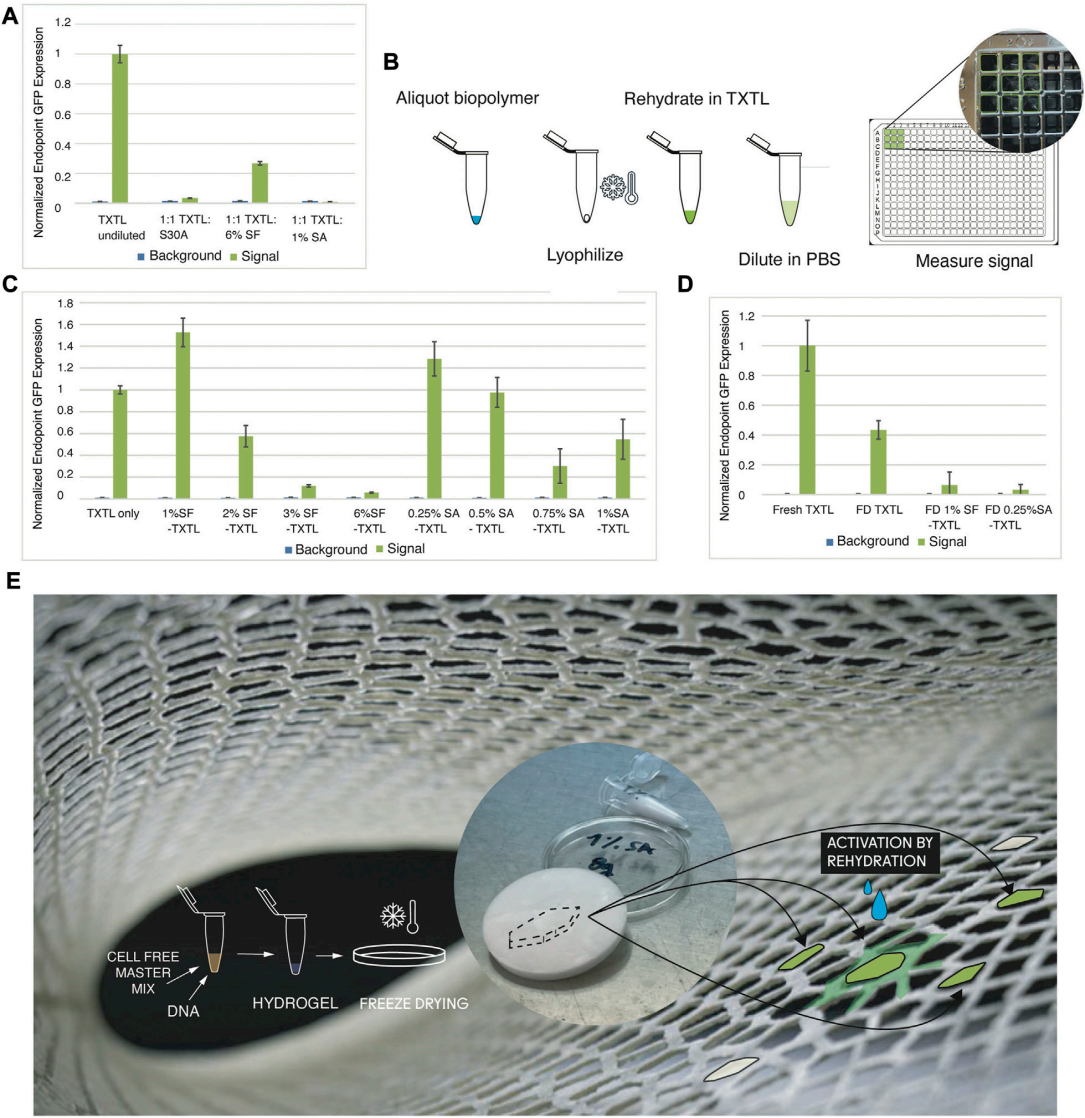
Microscale Design Methodology: TXTL Expression of GFP in Biopolymer Mixes. **(A)** Relative expression of TXTL when compared to 1:1 dilution in S30A Buffer, 6% SF, and 1% SF after incubation at 16H, 27°C. **(B)** To increase concentration of biomaterials without diluting TXTL mixes and overcome handling limitations of highly concentrated biomaterials, we lyophilize appropriate amounts and rehydrate in TXTL using 1.5 mL Eppendorf vials. 3 μL was expressed for 16H, 27°C, then diluted in 22 µL PBS and measured by transfer to a 384 well plate (with 3.65 mm diameter wells). **(C)** Using the method from **(E)** the effect on TXTL of SF from a range of 1%–6% and SA from a range of 0.25%–1% were tested. The highest expressing concentrations, 1% SF and 0.25% SA, were selected for subsequent experiments. **(D)** We tested the performance of lyophilized TXTL-1% SF and lyophilized TXTL-0.25% SA pellets after simple rehydration with water. **(E)** Envisioned and simplified design workflow to produce biosites in our future work consisting of TXTL protein expression in freeze dried bioblends embedded in robotically extruded structural lattices to be activated by hydration (background image is of printed lattice.

To test the TXTL activity in our biopolymer materials, we ran experiments diluting myTXTL in S30A buffer and comparing the GFP expression to dilution of TXTL in silk fibroin (SF) and in sodium alginate (SA). Figure 2A shows relative expression of undiluted TXTL when compared to 1:1 dilution in S30A Buffer, 6% SF, and 1% SA after incubation at 16H, 27°C. It appears that at our scaled down volumes, commercial kits are highly sensitive to dilution, where 98% of expression was lost after dilution in S30A buffer. Interestingly, dilution in silk fibroin in our tests resulted in lower expression loss at 74%. One reasonable hypothesis is that silk fibroin increases cell-free activity due to the macromolecular crowding effect, consistent with previous publications (Lee et al., 2020).

#### 3.1.2 Biopolymer—TXTL: Biopolymer lyophilization and re-hydration in TXTL

To avoid effects of dilution of TXTL and to overcome the handling limitations of higher concentrations of biopolymers, we developed a two-step workflow: first lyophilize the biopolymer solution separately and then rehydrate it in the TXTL mix (Figure 2B). We compare GFP expression after 16 h of incubation in 27°C for samples prepared with and without the lyophilization step, in which we tested undiluted TXTL volumes in low biopolymer concentrations by replacing DNA volume. The results show that lyophilization and rehydration do not appear to have a significant effect on expression. We used the lyophilization and rehydration method to test a range of concentrations of two biopolymers: silk fibroin (SF) and sodium alginate (SA). We chose our tested concentrations specifically within a range that was both structurally sound when lyophilized and workable at 1–10 µL volumes. At the lower end of this range (1% SF and 0.25% SA) are concentrations that were able to be lyophilized and still form structure, and at the higher end (6% SF and 1% SA), what was still liquid enough to pipette accurately at our small volumes. The results summarized in Figure 2C show that lower concentrations had a stronger effect in increasing expression, so 1% SF and 0.25% SA were used for the rest of our experiments.

Based on our results, we hypothesize that there is some optimal range of macromolecular crowding within a low concentration of added biopolymers that creates a positive effect on TXTL expression. It is reasonable to expect that like the entropically crowded environments of cells, there are some ideal conditions in which the total and by-species concentrations may positively affect the kinetic interactions of the reagents in TXTL. While not the focus of this paper, interesting directions using PEG molecules to crowd chromatin and studying the effects on gene expression are active areas of research (Pelletier et al., 2012).

#### 3.1.3 Biopolymer—TXTL: Lyophilization and re-hydration in water

We found that this increasing effect on expression did not carry over once TXTL was lyophilized together with our biopolymers (Figure 2D). Instead, a quantifiable but markedly decreased effect on the expression of protein was found in TXTL freeze-dried with both 0.25%SA and 1%SF pellets. For the latter, this is consistent with the results of Lee et al., 2020, which also found this decreased effect once SF was lyophilized with TXTL present.

As expected, we saw significant challenges to scaleup persisting upon blending materials with cell-free-protein expression systems that were designed to operate at, or close to, physiological conditions. Some of the expression activity remains as indicated by detectable GFP fluorescence when the background signal is subtracted. Others have seen similar (Ge et al., 2011; Lee et al., 2020) and we have formed several hypotheses involving the potential volume and entropic molecular crowding effects (Senske et al., 2014). For instance, under SEM analysis Lee et al. found that when cell free components were added to SF and lyophilized together, the material had collapsed as opposed to the spongy morphology without cell free added. We believe that a similar detrimental effect is occurring in our own experiments. Rather than the positive effect of low macromolecular crowding of materials in solution, the effect of lyophilizing biopolymer and TXTL together has likely resulted in overcrowding to a point in which expression is inhibited. However, testing these was beyond the scope of the current paper and would constitute important future research necessary to bring these types of materials from lab to market. We report qualitative activity as shown in Figure 2 that is clearly dependent on concentration, and substrate composition. We also found that evaporation, shelf life and cost of TXTL ingredients are important hurdles, each requiring a solution before industrial scale up can be practical.

We find that robustness and stability of expression are the main limitations for scaling up TXTL embedding. Rapid degradation may be attributed to the limitations of the food grade Harvest Right freeze dryer used for this step, which could not provide the ultra-low temperatures (<−80°C) that others have used). We also do not include a snap freezing step at −80°C due to similar limitations.

Outside the scope of this paper but as valid next steps testing other design strategies for incorporating biopolymers with TXTL would make the most sense. One experiment would be lyophilizing TXTL by itself and then rehydrating it in low biopolymer solutions as a potential work-around the negative effects of freeze drying TXTL and biopolymer together and possibly retaining the positive effect of expression in wet materials. More ambitious future work directions will likely build on developing custom cell-free systems in-house beyond the restrictions of the commercial TXTL mix (Sun et al., 2013), optimized to flexibly adjust components to the proper concentrations in bioblends. Developing an in-house custom-tailored TXTL system will also cut down the costs, which is important for testing and implementing the reactions in large-scale structures and add to the practicality of a business benefitting from a patentable formulation.

Figure 2E displays our proposed future workflow simplifying findings in Figure a-d towards streamlining to require only one freeze drying step. We envision TXTL protein expression in freeze-dried biopolymers as biosites. These biosites will be embedded in robotically extruded structural lattices and activated by hydration as described in Section 3.2 below.

### 3.2 Mesoscale Design Methodology

Biosites are embedded in robotically extruded structural lattices and distributed throughout them *via* rehydration and capillarity (Figure 1). Specifically, biosites are composed of 2 mm^2^ pellets which contain cell-free reactions with plasmid encoding GFP expression within silk fibroin or sodium alginate biopolymer solutions (Figure 1A). These composite pellets are freeze-dried into porous structures to be activated by simple rehydration. These final structures are about 1 cm^2^ and composed of at least five pellets (Figure 1C) within a non-active lyophilized biopolymer sponge (Figure 1B) providing mechanical support and attachment of active areas to lattice and ensuring good fluid distribution during rehydration (Figure 1D).

**FIGURE 3:**
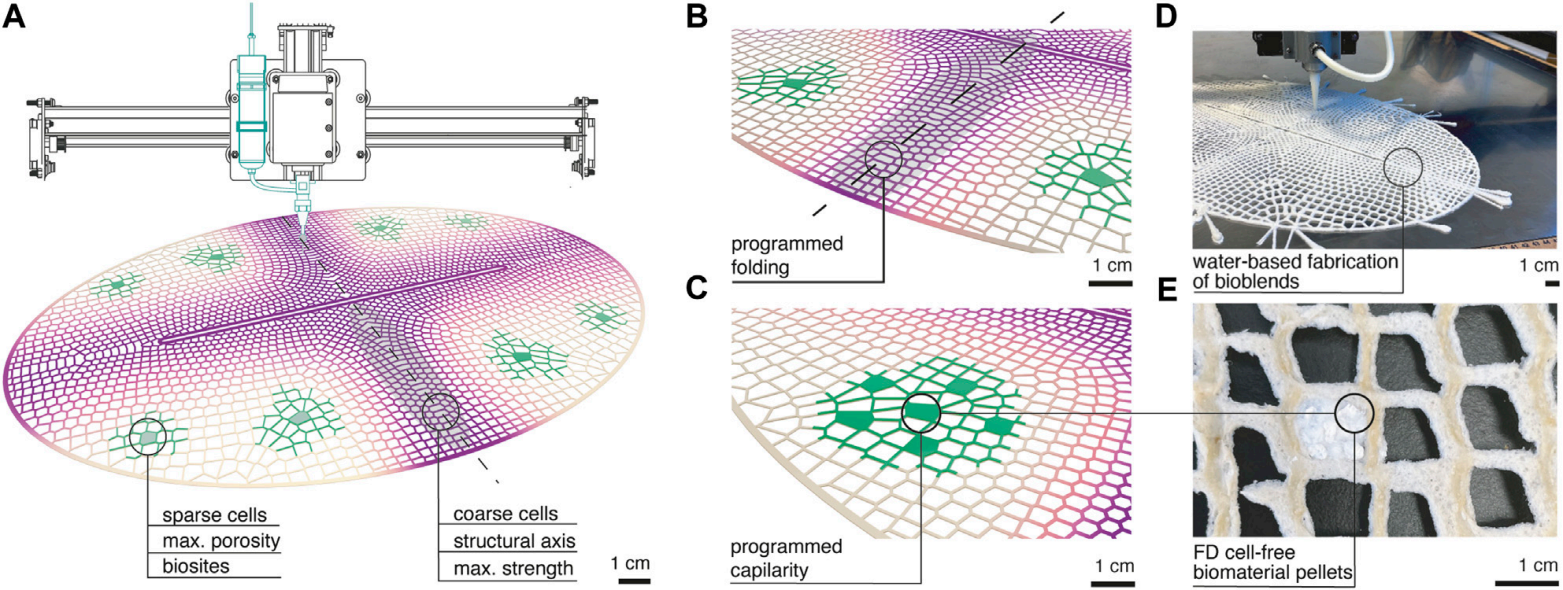
Mesoscale design methodology **(A)** Additive manufacturing is used to functionally distribute fibrous biopolymer blends in 50 cm long lattices with varied cell size and material composition. Geometrical design and material composition is graded throughout the lattice to target two distinct behaviors. Biosites are placed where maximum porosity material is laid and in presence of sparse cell areas ranging from 5 to 8 mm. Structural short axis of the oval is designed with coarse cell distribution of 1–4 mm and made by stronger material blends. **(B)** Programmed folding and strength in the structural axis is given by maximum shrinkage of the region because of higher amount of material per unit area and of higher concentration of silk fibroin in the blend. **(C)** Programmed capillarity is given by aerated and shorter fiber containing blends that improve porosity and fluid travel from biosite to lattice. **(D)** Image of functionally graded lattices being printed from bioblends. **(E)** Image of a biosite embedded in the printed lattice made of freeze-dried cell-free in sodium alginate biopolymer.

These composite arrangements can be included in designated bioactive zones within a lattice structure (Figure 1E). Lattices are additively manufactured from blends of cellulose fibers, chitosan gels, and silk fibroin solution (Figures 3A, E). The composition of bioblends through a lattice is varied according to the structural and biological performance required (Figure 3B). Porosity and strength are varied by adapting material composition within blends and varying parameters during synthesis. For instance, shorter cellulose fibers and high speeds providing aeration during blending are preferred for distribution around biosites. This generates maximum porosity in cured blends which improves bonding by wetting and providing fluid capillarity during rehydration of biosites (Figures 3B,D ). Higher concentration of silk fibroin provides stiffer dry areas which are desired in regions within lattices that are programmed to fold and hold attachment to other modules in the system (Figure 3C).

Defining fabrication strategies to realize these modular structures carrying environmentally interactive biosites, contributes to the contemporary vision of transferring synthetic biology’s potential beyond the field of regenerative medicine and towards design applications. Our work aligns with recent research where living textile structures allow detection of airborne pathogens and visualize their thread to human health within wearables (Nguyen et al., 2021), also living building components mimic trees with engineered cells that harness energy (for example, *via* photosynthesis) and assemble together molecules into large material structures that can redirect material and energy sources to adapt to an ever changing environment (Dade-Robertson, 2020), other examples create self-healing materials using embedded engineered cells (Dade-Robertson et al., 2015; Heveran et al., 2020; Caro-Astorga et al., 2021) and directed structural motifs assembled by embedded living cells responding to environmental cues such as humidity; pressure; presence of chemicals; light sources; etc (Gantenbein et al., 2022; McBee et al., 2022) evoking the potential of man-made structures to behave like living trees.

### 3.3 Macroscale Design Methodology

**FIGURE 4:**
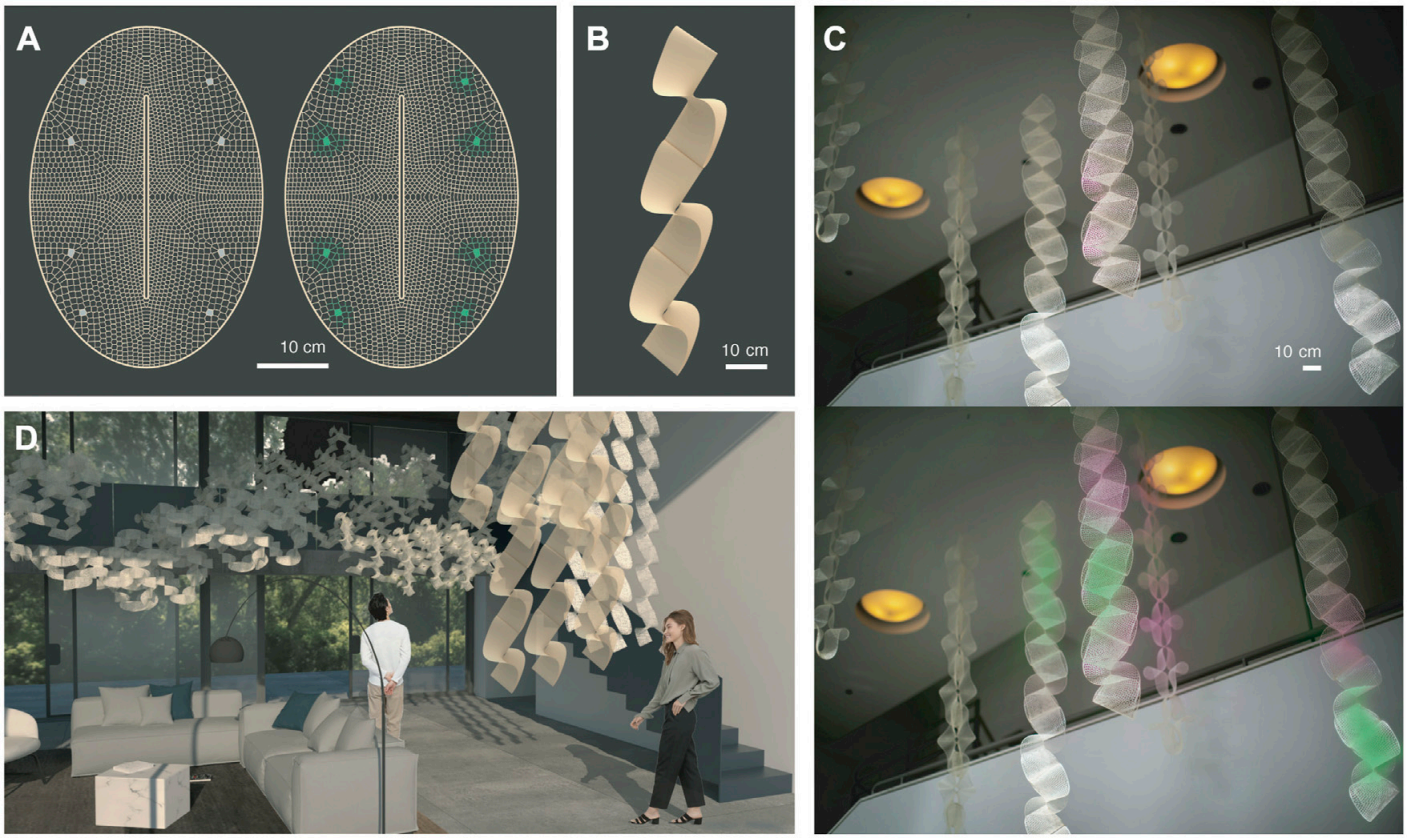
Macroscale Design Methodology. **(A)** Computationally designed functionally graded cell size distribution throughout lattices promotes folding, structural inertia, and attachment areas for biosites. **(B)** Geometries programmed also allow arrangement into ribbon structures able to hang from ceilings. **(C)** With potential to biologically sense and react to air in future work, ribbons provide adequate surface area and rotation to be integrated into indoor environments shown in **(D)**.

Global designs of lattices mentioned above were created by devising 2-dimensional structures able to transform into 3-dimension constructs (Figure 1G) and arrange into ribbon configurations (Figure 1H). We chose an oval global shape with a core longitudinal slit as it can undergo folding into a complex hypar-like geometry and so is able to occupy space and expose its surfaces to multiple directions for environmental interaction. The global shape discretized into lattice geometries was inspired by intricate mineral outer skeleton motifs of Collodaria radiolarian organisms (Suzuki and Not, 2015). We computationally designed functionally graded cell size distribution throughout the constructs. Importantly, shrinkage forces of these water-based systems are directly related to material accumulation. So, to promote self-folding and structural inertia for overall stability of the dry constructs, coarser cells ranging from 0.2 to 0.8 cm were distributed along the short axis of the oval (Figure 3B; Figure 4A). Conversely, we programmed sparser geometries with cell sizes superior to 0.8 cm in areas between short and long axis of the oval to obtain minimum curvature regions compatible with protein expression support. There, 1 cm^2^ freeze-dried systems could be easily attached (Figure 4A). Their exposure to the environment is maximized by oval folding (Figure 1G) and arrangement into ribbon structures able to hang from ceilings, which exposes maximum surface area and catches air flow at every orientation as it rotates (Figure 4B).

This is interesting for our ultimate goals of bioactive interaction with air (Figure 4D). We envision our structures to act as filters enhancing indoor air quality and providing stress-relieving aromas with bioactive sites able to sense air particles and react over time as drafted colorimetrically in Figure 4C. We note that odorants do not follow homogeneously diffusing paths, tendrils of scent are affected in their dynamics maximally by interactions with surfaces and air movements (McCaul et al., 2021) and so, linear and surface (as opposed to point-source) sensors and scent signal diffusers offer new modalities of study for the architecture of scent environments.

## 4 Discussion

Here we presented experimental results to contribute to the development of a multiscale design of biologically active (cell-free protein expressing) architectural structures. Our results show first steps towards modular multiscale design and fabrication of large structures with biologically active zones.

On the microscale, we show compatibility of the cell-free protein expression system (TXTL) within the biopolymer matrix of silk fibroin and sodium alginate using fluorescent protein GFP as a reporter. As future steps we consider the development of an in-house cell-free TXTL system to allow for custom-tailored components and cost reduction. On the mesoscale, we show the ability to tune material properties along additively manufactured lattice structures to support strength and provide porosity. This confers our structures with structural inertia and with signal distribution means from biosites. In ongoing developments towards airborne biointeraction these signals, currently fluorescence, may carry colorimetric sensing of air particles and aromatic response. On the macroscale, oval shapes with core slits that fold into paraboloids and arrange into ribbons are described and fabricated. Their lattice cell sizes are geometrically graded to induce folding of structures in denser motifs and to allow for biosite attachment in sparser motifs. Folding confers maximum surface area exposure of biosites as well as potential for modular assembly, which supports our goals of applying these biointeractive systems to air-interactive indoor partitioning.

### 4.1 Roadmap

**FIGURE 5:**
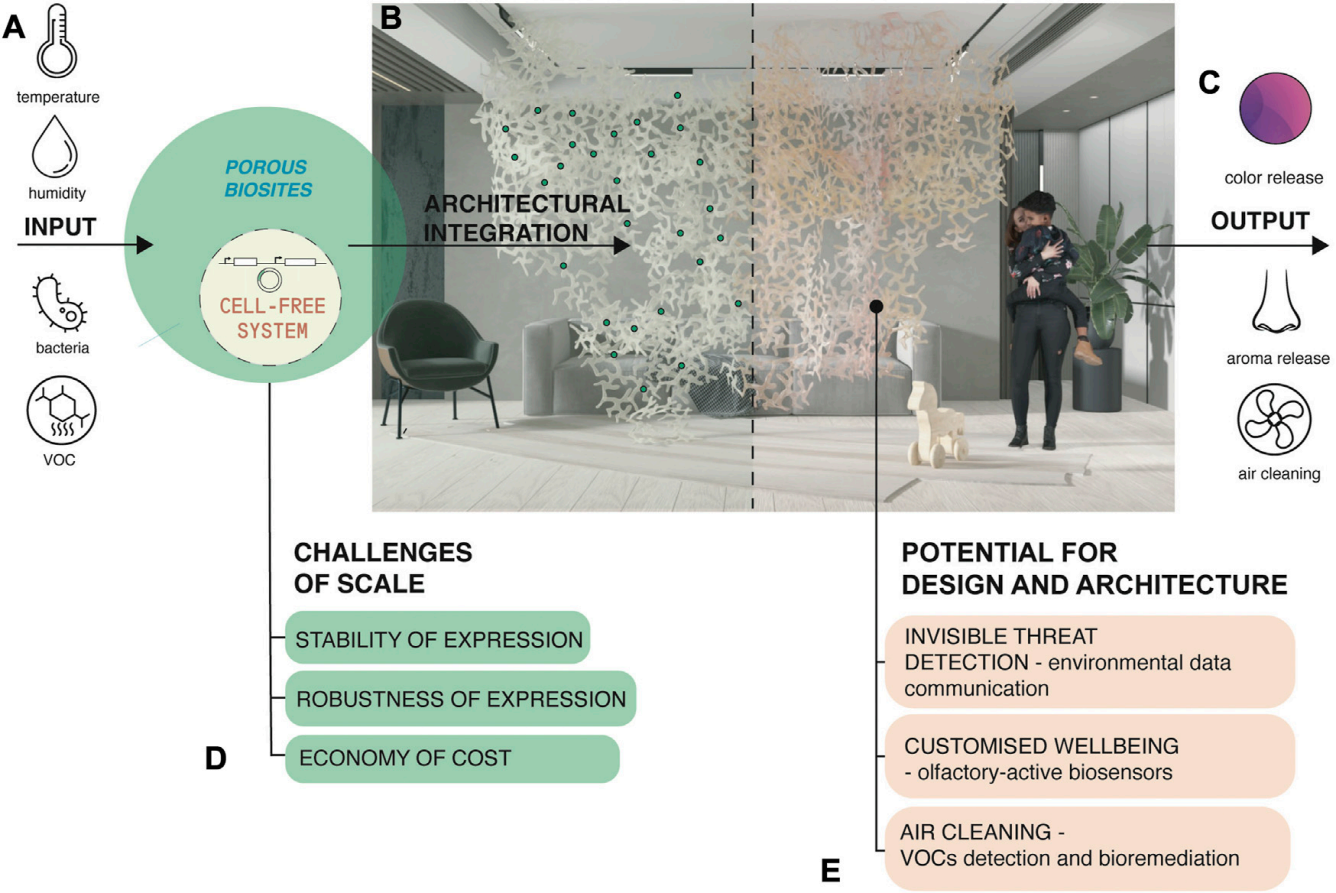
Scaling up cell-free systems for architectural solutions with embedded environmental responsiveness: a roadmap of potential and challenges. **(A)** Inputs into the envisioned system. **(B)** Biosites as porous bioactive zones within larger structures. Structures can be composed of multiple modules. **(C)** Outputs of the envisioned system. **(D)** Challenges to scaling up the technology for architectural and product applications. **(E)** Envisioned applications in design and architecture.

We envision a roadmap from the above experiments towards a range of architectural solutions enhancing health and wellbeing in indoor environments (Figure 5) potentially delving into the perceptual engineering and olfaction spaces as explained next. The inputs into our system are environmental parameters that biosites can sense and respond to. Input parameters include temperature, relative humidity, air particles such as toxic Volatile Organic Compounds (VOCs), aerosols, or pathogens (Figure 5A) (Nguyen et al., 2018; Moser et al., 2019; Nguyen et al., 2021; Gantenbein et al., 2022; McBee et al., 2022). The biosites are porous bioactive zones within our larger structure that contain cell-free systems embedded in a biopolymer matrix. Structures can be composed of multiple modules arranged into indoor partitions, wallpaper, or wall finishing systems, or conform to mechanical air flow devices in homes (Figure 5B). Cell-free systems within biosites are pre-programmed to respond to the mentioned parameters and activate a range of biochemical reactions. For example, an olfactory-active interior partitioning system could release active aromatherapeutic substances in response to detection of elevated levels of human stress. We note that olfactory sensing mechanisms borrowed from nature may include stabilized mammalian or insect olfactory receptors that have been shown to be expressible in a variety of cell-free systems, and their output readable in a variety of modalities from field-effect transistor settings to various spectroscopies (Mershin et al., 2015). Closing the loop biologically and recapitulating the functionality of an entire sensing and signaling pathway in a stabilized, cell-free system remains a challenge. Outputs of our system might include: color signals mapping the indoor air composition, release of odorants, neutralization and remediation of air pollutants and pathogens (Figure 5C).

Cell-free systems eliminate the need to maintain functions of living organisms. However, many challenges exist for scaling up this emerging technology for architectural and product applications (Figure 5D). One is robustness of expression by developing detectable and reproducible cell free expression in response to well-characterized stimuli. Another challenge is stability of expression by developing long shelf-life in between cycles per application (Sato et al., 2022). Both robustness and stability of expression can reduce the cost of current commercial TXTL cell-free systems, for that, improvements can include; adding stabilizing additives, revision of lyophilization-to-dehydration sequences, development of new protein expression reagent mixes, or producing homemade TXTL, to deploy scaled production. Finally, our envisioned applications in design and architecture include (Figure 5E); (1) environmental data communication and invisible threat detection such as airborne threat detection (in olfactory sensing mode) and pest repellent (in olfactory emitting mode), (2) olfactory-active biosensors detecting and correcting aromas that can act to reduce stress levels, improve health of indoor spaces, and the wellbeing of its inhabitants, (3) air cleaning purposes by detecting and trapping VOCs and releasing pollution-remediating compounds in response. We consider a world where these systems can be embedded into wall dividers, tiles, ceiling and floor partitions, or everyday consumer objects.

Our results demonstrate the potential and challenges involved in practically deploying cell-free protein expression-based interactive components in printed materials and objects with variable robustness of expression and durability. Here we report first steps towards building materials that host biologically active zones within lightweight, porous, and foldable lattices printed from biological material blends. We anticipate novel applications for this multiscale platform in product design and architecture to emerge once stability and scaling up strategies are developed to address expression level and durability issues.

## Data Availability

The original contributions presented in the study are included in the article/supplementary material, further inquiries can be directed to the corresponding authors.
